# Recent Advances and Future Prospects of Mycosporine-like Amino Acids

**DOI:** 10.3390/molecules28145588

**Published:** 2023-07-22

**Authors:** Jiahui Peng, Fangyu Guo, Sishi Liu, Haiyan Fang, Zhenshang Xu, Ting Wang

**Affiliations:** 1 State Key Laboratory of Biobased Material and Green Papermaking, Qilu University of Technology, Shandong Academy of Science, Jinan 250353, China; 18668900804l@sina.com (J.P.); gfangyu2022@163.com (F.G.); 17520326387@163.com (S.L.); fhy@qlu.edu.cn (H.F.); 2 School of Bioengineering, Qilu University of Technology, Shandong Academy of Science, Jinan 250353, China

**Keywords:** mycosporine-like amino acids, extraction and identification, heterologous expression, occurrence and distribution, biosynthesis, physiological function

## Abstract

Mycosporine-like amino acids (MAAs) are a class of water-soluble active substances produced by various aquatic organisms. However, due to the limitations of low accumulation of MAAs in organisms, the cumbersome extraction process, difficult identification, and high cost, MAAs have not yet been widely used in human life. Recently, there has been an emergence of heterologous synthesis for MAAs, making increasing yield the key to the quantification and application of MAAs. This review summarizes the latest research progress of MAAs, including: (1) introducing the biodistribution of MAAs and the content differences among different species to provide a reference for the selection of research subjects; (2) elaborating the species and molecular information of MAAs; (3) dissecting the synthesis mechanism and sorting out the synthesis pathways of various MAAs; (4) summarizing the methods of extraction and identification, summarizing the advantages and disadvantages, and providing a reference for the optimization of extraction protocols; (5) examining the heterologous synthesis method; and (6) summarizing the physiological functions of MAAs. This paper comprehensively updates the latest research status of MAAs and the various problems that need to be addressed, especially emphasizing the potential advantages of heterologous synthesis in the future production of MAAs.

## 1. Introduction

**Figure 1:**
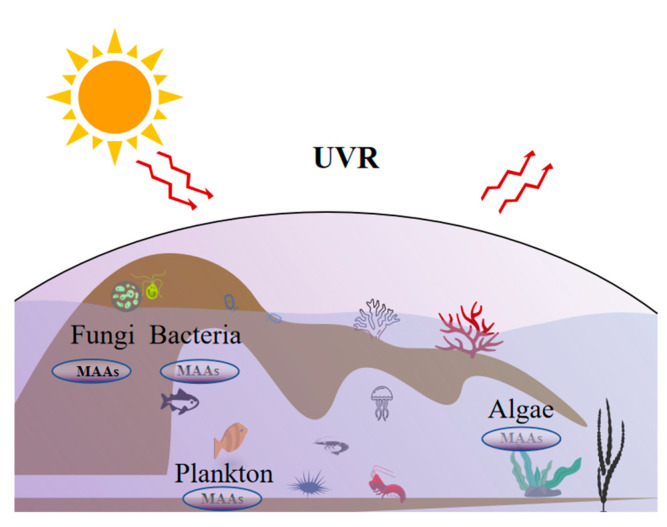
Biodistribution of MAAs derived from algae, bacteria, fungi, and plankton.

The increasing severity of the ozone hole problem has led to a greater amount of ultraviolet radiation (UVR) reaching the ground and water bodies, resulting in photoaging of the skin and inducing numerous diseases. This emphasizes the importance of searching for naturally derived photoprotective compounds from diverse organisms to counteract the damage caused by UVR [1,2,3]. Marine macroalgae, which have been exposed to ultraviolet-A (UVA, 315–400 nm) and ultraviolet-B (UVB, 280–315 nm) radiation since the dawn of life on Earth, have developed various defense systems such as avoidance, DNA repair, and cellular protection against UVR through the production of carotenoids and mycosporine-like amino acids (MAAs) [4]. MAAs will have an unassailable position in the sunscreen market in the future, due to their numerous biological activities, which include anti-UV, anti-inflammatory, anti-cancer, and anti-aging properties, as well as their role in promoting cell proliferation, wound healing, and the protection of DNA from damage [5]. In addition to algae, bacteria, fungi, and plankton have also evolved the ability to synthesize, accumulate, and metabolize MAAs [6,7,8,9,10,11] (Figure 1).

Environmental factors such as UVR, pH, temperature, and salinity greatly influence the accumulation of macroalgal-derived metabolites (MAAs) [12,13]. Studies have found that various macroalgae can produce one or more MAAs, and their composition varies based on distribution [14,15,16,17,18]. Moreover, due to the different absorption maxima of these MAAs, they often co-exist and form an effective photoprotective filter [19]. Thus, MAAs are widely researched for their anti-UV properties. Nevertheless, they also have other beneficial effects such as anti-inflammatory, anti-cancer, cell growth promotion, DNA damage protection, and wound healing properties [20,21], which can be harnessed for pharmaceutical applications. To maximize the potential of MAAs, it is essential to have a thorough understanding of their chemical structure and activity, as well as their biosynthetic pathways. This will enable the development and commercialization of MAAs for early and large-scale applications in various fields such as pharmaceuticals, medicine, and biotechnology.

MAAs are obtained almost exclusively from algae by in vitro extraction [22,23,24,25]. Despite wide distribution in aquatic organisms, the low content and excellent water solubility of MAAs poses a significant challenge for extraction. To maximize extraction rates, it is essential to invest in research of efficient isolation and purification methods. Moreover, long growth periods and high cost of fishing limit the use of MAAs, and optimization of their production and concentration in organisms, including genetically modified organisms, is key to increasing their applicability. Alternatives, such as heterologous expression or organic synthesis of analogs, should also be explored [26,27]. However, the chiral center of MAA compounds makes them difficult to synthesize chemically and the scarcity of synthetic feedstock means that large-scale synthesis remains a challenge. MAAs are beginning to gain attraction in heterologous production and the future of MAA applications may be unlocked through low-cost, high-rate heterologous synthesis.

## 2. Biodistribution of MAAs

### 2.1. Algae

The study of MAAs in marine algae began to be extensively explored in the 1990s. Currently, marine macroalgae are the most abundant source of MAAs, though their content varies. To date, MAAs have been identified in 572 marine macroalgae [28], including a wide array of species such as green algae, brown algae, red algae, and cyanobacteria [29,30]. Red algae have been found to possess the highest concentration of MAAs and the most abundant species of MAAs. Among the 572 species of macroalgae containing MAAs, red algae accounted for 486 species, and their MAAs contained not only common types such as shinorine, porphyra-334, mycosporine-glycine, asterina-330, and palythine, but also aplysiapaly-thine B, usujirene, collemin A, and other types of MAAs. In particular, *Rhodymenia* spp. had a surprisingly high MAA content (8.8–142.9 mg/g DW, average value 75.85 mg/g DW) [28]. Microalgae, known for their rapid growth and easy cultivation, have also been used as a partial production source of MAAs [31]. However, the content of MAAs in green and brown algae is usually much lower, ranging from 0.2 to 0.275 mg/g DW. MAAs found in cyanobacteria were the first to be researched, with shinorine and porphyra-334 being the most predominant MAAs in marine cyanobacteria [32,33,34]. Shinorine can be extracted from *Aphanizomenon flos-aquae* CHAB5921 at concentrations of up to 0.385 μg/mg [35], and porphyra-334, produced by *Nostoc verrucosum*, accounts for more than 90% of the total MAAs [36]. Thus, the appropriate algae can be chosen to extract different species of MAAs according to the desired requirements.

In addition to cyanobacteria, red algae have been extensively studied for their abundance of various MAAs [25,37,38,39,40,41]. Maria et al. [37] identified 11 crude extracts of MAAs from three different sources of red algae. The most abundant compounds in the extracts were shinorine, palythine, asterina-330, and porphyra-334. Similarly, Sun [23] also found high concentrations of shinorine, palythinine, and porphyra-334 in the extracts of four macroalgae when extracting MAAs. By analyzing the pigment content and MAA composition of macroalgae, it was discovered that red algae with photoprotective pigments and various MAA species can better survive under high-level radiation environments. This also indicates the strong UVR and photoprotective ability of MAAs. Furthermore, the ability of eukaryotic algae to produce a wide range of MAAs suggests the evolutionary diversity of MAA biosynthetic pathways in photosynthetic eukaryotic cells.

### 2.2. Bacteria and Fungi

Originally isolated from fungi by Leach, a variety of compounds with the same basic chemical structure as MAAs have since been identified from the mycelium of fungal spores. MAAs contain a proprietary oxygenated complex-carbonyl chromophore (oxo-MAAs), while certain cyanobacteria are characterized by imino-carbonyl structures (imino-MAAs), suggesting an evolutionary segregation of their synthesis between fungi and cyanobacteria. In 2007, the biosynthetic pathways and molecular structures of various MAAs present in fungi were reported in detail in the database of MAAs established by Sinha et al. Subsequently, genome mining identified two microbial strains of Actinomycetales, which were hypothesized to have gene clusters homologous to cyanobacterial biosynthetic gene clusters. *Pseudonocardia* sp. strain P1 was found to accumulate small amounts of MAAs compounds, while no MAAs were observed in the culture of *Actinosynnema mirum* DSM 43827, suggesting that this biosynthetic gene cluster may not be expressed in this microorganism [27]. Mycosporines have also been reported to be synthesized in yeast and many of the substances synthesized by freshwater yeasts grown under photo-synthetically active radiation (PAR) have a maximum absorption peak at 309–310 nm, which is consistent with the characteristics of MAAs [42]. Further, 25 yeast strains capable of producing mycosporines were isolated from the natural environment of Argentina, thus laying the foundation for screening strains that could produce MAAs. The consistent occurrence of mycosporine in certain phylogenetic taxa suggests that this property has evolutionary significance, while the presence or absence of mycosporine may have potential applications in yeast systematics [43].

### 2.3. Plankton

In 1977, Ito and Irata first isolated and characterized mycosporine-glycine from the tropical animal *Palythoa tuberculosa*, while a phytoplankton photoprotective compound database was established in 2000 [14]. Generally, the types of MAAs produced by plankton include mycosporine-glycine, palythine, asterina-330, shinorine, and porphyra-334 [7]. Hylander detected MAAs in 43 species of zooplankton, including rotifers, daphnia, krill, copepods, and branchiopods, as well as sea urchins and fish [7,44]. Moreover, MAAs have been found in sea anemones, jellyfish, corals, sea stars, and flatworms, and it is likely that they originate from their diets [6]. Among these organisms, *Daphnia* spp. contain high concentrations of MAAs, while only traces are found in branchiopods and krill; such trace amounts of MAAs may also come from ingested food.

MAAs in organisms are tightly correlated with multiple factors, the most important of which being exposure to UVR [13]. Ciliates from alpine lakes exposed to high UVR levels were observed to contain seven MAAs, while ciliates from lakes with lower UV intensity did not contain any MAAs [44]. In addition, factors such as temperature, initial MAA concentration, and others [45] are involved in the synthesis of MAAs. For example, some zooplankton have been found to contain higher MAA concentrations in extreme environments such as those with high salinity and high radiation [6,46]. Furthermore, a regular seasonal variation in MAA concentrations has been observed in phytoplankton and copepods, with MAA concentrations being 3.6-fold and 3.0-fold, respectively, higher in the summer compared to the cold period [47]. Several studies [27,47,48,49,50] agree that during periods of elevated environmental stress, some zooplankton and phytoplankton produce high concentrations of MAAs, which play an important role in protecting aquatic organisms from UV damage. This subsection further elucidates the species diversity and abundance of MAAs from a zooplankton perspective, providing a potential source for large-scale production of MAAs.

MAAs can be synthesized by a variety of organisms, such as algae, bacteria, zooplankton, fungi, and plants. Three mechanisms are involved in their synthesis and accumulation in various organisms: (1) MAAs are synthesized by the organisms themselves and their content is not affected by light; (2) organisms possess self-protective mechanisms developed during evolution that induce rapid MAA synthesis when exposed to UVR; and (3) some aquatic organisms lack MAAs and obtain them through dietary intake. While bacteria and fungi can accumulate small amounts of MAAs, the concentration of MAAs in plankton tends to increase with altitude but stops at a certain limit. Algae, particularly cyanobacteria, green algae, and red macroalgae, are rich sources of MAAs and provide a plethora of raw materials for research. However, there is a need to cultivate algae sustainably to optimize MAA yields.

## 3. Properties and Synthesis of MAAs

### 3.1. Molecular Structure of MAAs

**Figure 2:**
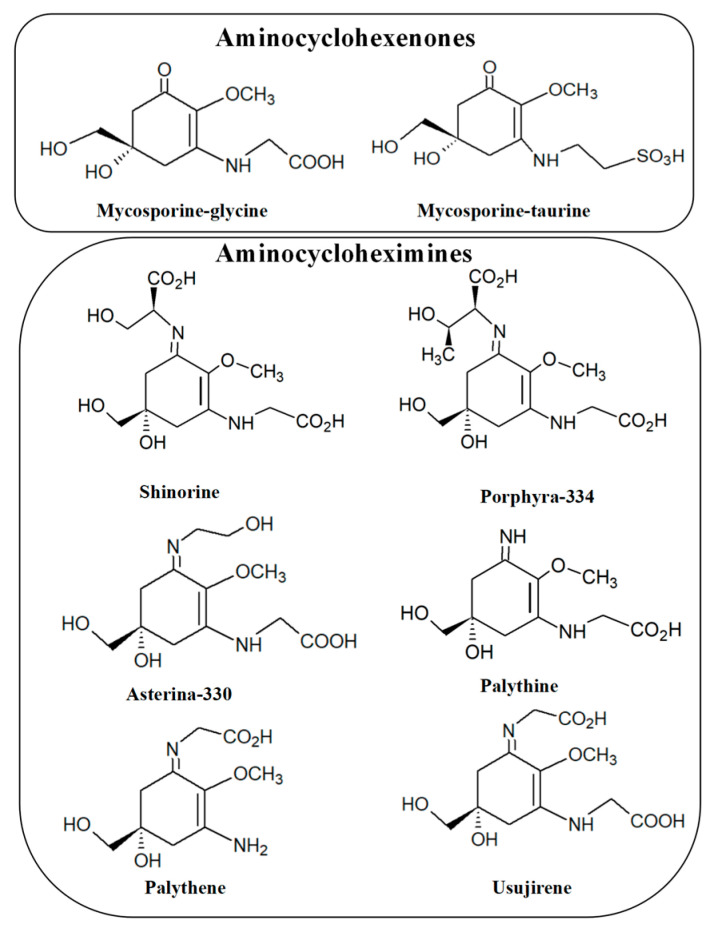
Chemical structures of some MAAs.

MAAs are low-molecular-weight compounds (<400 Da) consisting of aminocyclohexenones and aminocycloheximines bearing nitrogen substituents [27,51,52]. The aminocyclohexenones group contains an aminocyclohexenone and an amino acid and presently comprises two main species: mycosporine-glycine and mycosporine-taurine [53]. These are the only known aminocyclohexenones of marine origin. Aminocycloheximines, on the other hand, are cyclohexenimines with one amino acid, methylamine, or amino alcohol attached to the third carbon atom, while an enaminone chromophore is attached to the first carbon atom [54]. Usually, a glycine substituent is found at the third carbon atom of the MAAs’ ring system, although glycosidic bonds and sulfate esters may also be part of the imine group of certain MAAs [55]. Compared to cyclohexenone MAAs, imines are more diverse and include porphyra-334, shinorine, mycosporine-2-glycine, palythinol, palythene, etc. [19,31,56] (Figure 2).

MAAs have a maximum absorption range of 268–362 nm, which is mainly concentrated between 310 and 360 nm [57]. This range is attributed to the presence of conjugated double bonds in their molecules, causing an absorption peak that ranges from 310 nm for the cyclohexenone structure to 360 nm for the cyclohexylamine structure [52]. Differences in their absorption spectra are largely due to variations in side groups and nitrogen substituents [58]. Generally, mycosporine-glycine has a maximum absorption wavelength of 310 nm, palythine 320 nm, and shinorine 334 nm [52]. MAAs have high molecular absorbance and can absorb both UVA and UVB radiation [54]. Their ability to protect a wide range of UVR wavelengths is further enhanced by the presence of functional groups, such as carboxyl and hydroxyl groups, and substituents, such as methyl groups, which also render them water- and alcohol-soluble, thus giving them their unique biological activity.

### 3.2. Types of MAAs

MAAs were first reported in 1960 and, since then, more than five hundred species have been identified to contain MAAs or their constituents [28]. Despite the long-standing presence of MAAs, there are few resources available to summarize the number of MAAs that have been identified over the decades. Therefore, we have targeted the species and molecular information of the MAAs identified so far, as illustrated in Table 1.

### 3.3. Biosynthesis Pathways of MAAs

Early organisms produced MAAs in order to circumvent the damaging effects of high-intensity UVR, thus their evolution [85]. Cyanobacteria were the first to produce MAAs, with biosynthesis occurring within hours of UVR exposure. Studies of MAAs’ biotransformation in marine algae revealed that their synthesis occurs through the pentose phosphate pathway (PP pathway) [86], with sedoheptulose 7-phosphate (SH-7P) forming 4-deoxygadusol (4-DG) as its parent core structure through the transformation of enzymes 2-epi-5-epivaliolone synthase (EVS) and O-methyltransferase (OMT) [87]. Different amino acids can condense with aminocyclohexenones to form a variety of MAAs. For instance, 4-DG and glycine binding in the presence of ATP-ligase generates mycosporine-glycine [86,88,89], the first MAAs to be transformed by amino acid substitution [54,90]. Serine binding to mycosporine-glycine via nonribosomal peptide synthetase (NRPS) or D-alanyl-D-alanine ligase (D-ala-D-ala-ligase) leads to the formation of shinorine.

The deletion of the cyclase EVS gene in *Anabaena variabilis* ATCC 29413 had little effect on the production of MAAs [87], suggesting that the biosynthesis of MAAs is not exclusive to the PP pathway. MAAs’ biosynthesis was further demonstrated to occur through the shikimate pathway by the addition of inhibitors. Proteomic data showed that the shikimate pathway is the major route for UV-induced MAAs biosynthesis. 3-Deoxy-d-arabinoheptulosonate-7-phosphate (DAHP) synthase catalyzes the first step of the shikimate pathway [91]. Genes encoding enzymes of the shikimate pathway have been identified in the genome of sea anemones, including a 3-dehydroquinate synthase (DHQS) adjacent to O-MT [92]. This indicates that DHQS is also involved in the biosynthesis of MAAs [93]. Both the PP pathway and the shikimate pathway are inextricably linked to MAAs’ biosynthesis [61,86]. Evidence suggests that the shikimate pathway is the predominant pathway for providing photoprotection, whereas MAAs produced by the PP pathway are likely to have other biological functions [89].

**Figure 3:**
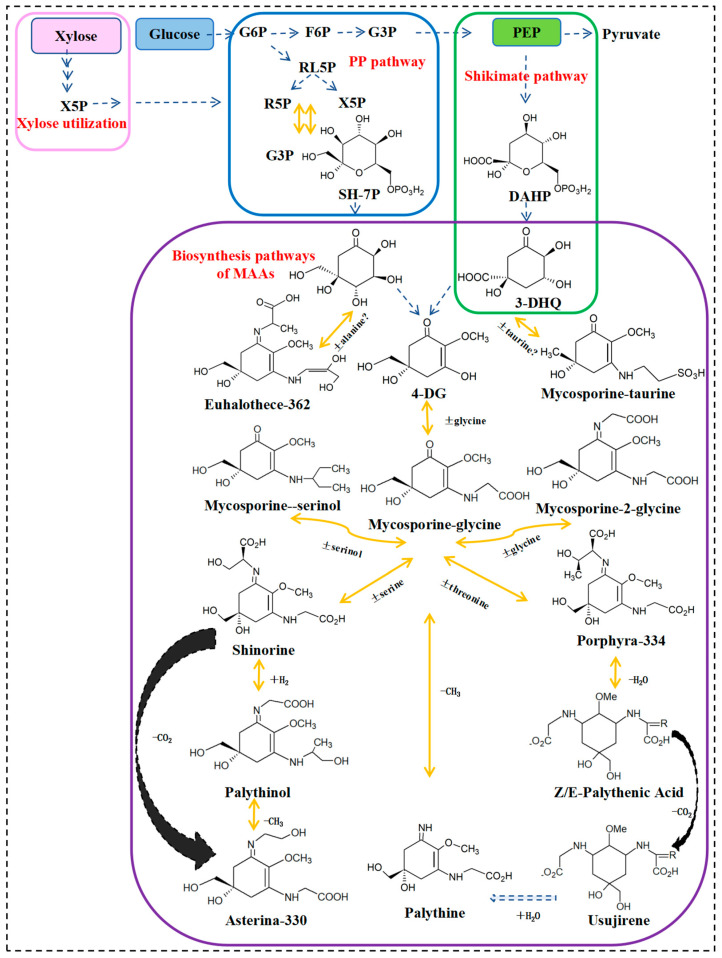
MAAs’ biosynthetic pathway.

Figure 3 depicts the biosynthetic pathway of MAAs. Other MAAs are synthesized through the modification of side chain groups, such as esterification, amidation, dehydration, decarboxylation, hydroxylation, sulfonation, and glycosylation, as well as through nitrogen substitution.

## 4. Acquisition and Detection of MAAs

### 4.1. Extraction In Vitro

The majority of binary solvent mixtures can be used for the isocratic elution of UV-absorbing compounds in HPLC, such as water, ethanol, methanol, and acetonitrile [23,52,94,95,96], and more widely for the separation of MAAs by gradient elution [97]. Volkmann M [22] proposed a widely applicable method for the extraction of MAAs: using a 0.5% methanol and 0.2% acetic acid–water solution, the sample was extracted at 4 °C with shaking for 12 h and then filtered through a 0.2 um filter membrane. This method is not only applicable to algae, but also to the extraction of MAAs from terrestrial and freshwater organisms. 0.1% acetic acid in methanol–water (25:75) solution has also been used for the isolation of MAAs from marine organisms [98]. When the extraction solvent was 25% methanol or 25% ethanol, the extraction yields of MAAs from four red macroalgae using 25% ethanol and distilled water revealed yields of 9.01–12.39% and 19.99–32.34%, respectively; however, the MAA extraction yield of *Gracilaria confides* was low [23]. This suggests that the extraction rate is affected by different extraction conditions and algal species. Additionally, the feed-to-liquid ratio, extraction time, and extraction level were found to have similar effects on the yield of MAA extracts of the four red algae; the yield of MAA extracts increased with increasing levels of the variable factors, but started to decrease after reaching the maximum MAA extraction rate. Zwerger M [25] used water, ethanol, ammonium sulfate, and methanol in ascending mode as an aqueous two-phase system, which enabled the separation of two MAAs from algal crude extracts within 90 min. Solid-phase extraction was then used as the second purification step; 15.7 mg of high-purity shinorine and 36.2 mg of porphyra-334 were isolated from the crude extract of seaweed, and a rapid centrifugal partition chromatographic method for MAAs was developed. This fast centrifugal partition chromatography method can quickly isolate MAAs with high purity but the extraction solvent system often requires precise experiments for successful determination.

However, due to their high solubility in mobile phases used for liquid phase analysis, such as methanol, acetonitrile, and aqueous solutions, obtaining high-purity MAAs via in vitro extraction and separation is difficult. Moreover, MAAs vary between different species of algae, making the extraction and separation process even more complicated. Furthermore, the solubility and absorbance maximum (λmax) properties of MAAs depend on the polarity and pH of the extraction solvent system, making it hard to distinguish and separate them. During the extraction process, there is a risk of irreversible loss of the target product. As a novel active substance with commercial potential, the current progress in extraction, isolation, and characterization of MAAs is inadequate in terms of applicability. Therefore, if MAA accumulation in organisms cannot be increased, there is a need to investigate more efficient extraction and separation methods, which are essential for the extensive development and wide application of MAAs.

### 4.2. Heterosynthesis

Heterologous synthesis is an effective method for the production of natural products of varied origins [99,100]. Extraction of MAAs from natural sources is highly inefficient, and aquaculture methods to obtain them are costly and require a prolonged cycle. Chemical synthesis, on the other hand, is extremely costly and has long cycle times. Additionally, the refractory issues associated with chemical synthesis further impede access to MAAs in a sustainable manner [101]. Heterologous synthesis of microbial natural product biosynthetic pathways, combined with advanced DNA engineering, offers numerous advantages including shorter timeframes, reduced costs, and improved product yields. In vitro extraction methods, although able to yield trace amounts of MAAs, do not provide commercially available MAA standards; consequently, the production of MAAs through the utilization of heterologous hosts is highly desirable [102].

Hosts commonly utilized for the heterologous production of MAAs include *Escherichia coli*, *Streptomyces*, and *Saccharomyces cerevisiae* [26,27,83,86,103,104] (Table 2). Gao et al. [103] introduced the biosynthetic gene for Candida punctata MAAs into *E. coli* and successfully expressed and produced the precursor mycosporine-glycine. The additional overexpression of the mysD gene facilitated the conversion of mycosporine-glycine to shinorine, mycosporine-2-glycine, and porphyra-334, thus elucidating a novel model of enzymatic synthesis of imipramine. To enhance the yield, Jin et al. [88] introduced four biosynthetic genes for shinorine from the cyanobacterium *Nostoc punctiforme*. They first increased the accumulation of SH-7P using xylose as a co-substrate, then suppressed glucose catabolism through the glycolytic pathway, and finally deleted the HXK2 gene encoding hexose kinase. This ultimately resulted in a *Saccharomyces cerevisiae* strain producing 68.4 mg/L of shinorine. Consequently, fermentation technology presents a strong competitive advantage for the production of MAAs.

Whereas in vitro extraction route for obtaining MAAs has been less studied for heterologous synthesis, research on the heterologous synthesis of MAAs in the past decade is still scarce. Despite the progress made in the heterologous synthesis of MAAs, the expression levels remain low, which is directly attributed to the limitations of the host used for heterologous production. Due to a significantly different genetic background from cyanobacteria, such as gene GC content, promoter, and structure of RNA polymerase [104,105], *E. coli* is limited in its ability to express cyanobacterial natural products, resulting in low or no yield [106]. Additionally, expression competition among external gene clusters can complicate the isolation and identification of expressed natural products. A combination of transcriptional and metabolic analysis can now be used to identify rate-limiting steps in the heterologous production of MAAs, while genetic modification can expand production. Additionally, the addition of multiple promoters can lead to increased production [106]. In the future, efforts should be made to discover more biogenic clusters of MAAs and optimal production hosts in order to optimize production and increase yields. Compared to extracting MAAs from organisms with low accumulation, we see great potential for heterologous synthesis of MAAs through gene editing, etc.

**Table 2:** Gene clusters and major products of MAAs biosynthesis from different sources.

Species	Strain Number	Gene Cluster	Host	Product	Yield	Reference
*Cyanobacteria*						
*Anabaena variabilis*	ATCC 29413	ava_3858	*E. coli*	Shinorine	/	[86]
		ava_3857				
		ava_3856				
		ava_3857				
*Nostoc punctiforme*	ATCC 29133	npun_R5600	*E. coli*	Mycosporine-2-glycine, Shinorine, Porphyra-334	/	[103]
		npun_R5599				
		npun_R5598				
		npun_R5597				
*Fischerella sp.*	PCC9339	RS0129515	*Synechocystis* sp. PCC6803	Shinorine	2.37 ± 0.21 mg/g DW	[106]
		RS0129520				
		RS0129525				
		RS0129530				
*Nostoc linkia*	NIES-25	NIES25_64130	*E. coli*	Mycosporine-glycine, Palythine-serine, 4-Deoxygadusol, Shinorine, Porphyra-334	/	[26]
		NIES25_64140				
		NIES25_64150				
		NIES25_64160				
*Cylindrospermum stagnale*	PCC 7417	MylA	*E. coli*	Mycosporine-ornithine, Mycosporine-lysine	/	[73]
		MylB				
		MylC				
		MylD				
*Scytonema* cf. *Crispum*	UCFS15	UCFS15_00409	*E. coli*	Mycosporine-glycine, Shinorine	/	[83]
		UCFS15_00408				
		UCFS15_00407				
		UCFS15_00406				
*Scytonema* cf. *Crispum*	UCFS10	UCFS10_04336	*E. coli*	Mycosporine-glycine, Shinorine	/	[83]
		UCFS10_04337				
		UCFS10_04338				
		UCFS10_04339				
*Fungi*						
*Aspergillus clavatus*	NRRL 1	acla_55850	/			[27]
		acla_55840				
		acla_55830				
*Aspergillus nidulans*	FGSC A4	an6403.2	/			[27]
		an6402.2				
*Cnidarians*						
*Nematostella vectensis*	/	nemvedraft_v1g70416	/			[27]
		nemvedraft_v1g167288				
		nemvedraft_v1g206757				
*Dinoflagellata*						
*Heterocapsa triquetra*	/	DQ517901 (MRNA)	/			[27]
*Actinobacteria*						
*Actinosynnema mirum*	DSM 43827	amir_4259	*Saccharomyces cerevisiae*	Shinorine	54 mg/L	[107]
		amir_4258				
		amir_4257				
		amir_4256				
*Pseudonocardia* sp.	P1	pseP1_010100031440	/			[13,108]
		pseP1_010100031435				
		pseP1_010100031430				
		pseP1_010100031425				

*/* No relevant information.

### 4.3. Detection of MAAs

Influenced by the number and position of conjugated double bonds in the chemical structure as well as the reactive substituent groups on their side chains, MAAs show strong UVR absorption peaks between 310–360 nm, making ultraviolet spectroscopy (UVS) an effective way to detect their presence in samples. Persaud [109] has used UVS to detect MAAs in copepods in lakes of varying UVR intensities. Additionally, infrared spectroscopy (IRS) has been used for qualitative and quantitative studies of different types of natural products, demonstrating its validity and simplicity [110,111]. Nuclear magnetic resonance spectroscopy (NMR) is also capable of structural identification of MAAs, but has low sensitivity and requires more complex sample preparation [112]. Similarly, gas chromatography–mass spectrometry (GC/mass spectrometry) also has detection disadvantages, such as the need for MAA samples to be easily vaporized and the fact that MAA samples can only be determined in organic solutions.

Currently, the most common methods for the detection and quantification of MAAs are high-performance liquid chromatography (HPLC), based on their retention times and absorption maxima, and liquid chromatography coupled with liquid chromatography–electrospray ionization mass spectrometry (LC-ESI-MS), which enables examination of the structural diversity of MAAs. For example, mycosporine-glycine, palythine, and palythinol were successfully separated by HPLC from a Pacific stony coral sample [60]. Moreover, the combined use of multiple instruments may be more effective for the detection and structure determination of MAAs. Chollet-Krugler M validated the quantitative method for the determination of MAAs in lichen specimens using HPLC-DAD [113]. Suh SS used HPLC to isolate three MAAs from seaweed extracts and subsequently confirmed their specific structures by triple quadrupole mass spectrometer (TQMS) [114]. LC-ESI-MS can also be used to determine the composition and proportion of all MAAs extracted from certain macroalgae [23]. Additionally, a diode array detector (DAD) can be employed for UV scanning to identify MAAs; however, this process can be challenging due to the presence of unknown, closely related compounds with similar absorption maxima and retention times. To address this issue, Orfanoudaki M [37] used fast centrifugal partition chromatography to analyze extracted MAAs samples and, by combining the use of UVS, NMR, and LC-MS in comparison with published data, developed a novel method for the detection of MAAs in greater detail. This method was also used to refine the purification step and achieve a high extraction yield.

Therefore, an increasing number of researchers are studying MAAs using multiple spectrometers (Table 3; [58,113,115,116,117,118]). To date, MAAs have been identified and characterized by various experimental techniques, such as UVR, IRS, GC/MS, NMR, LC-ESI-MS, LC-MS, TQMS, and DAD. Purity is a paramount factor in conducting all scientific research and tests, and these analytical techniques are often combined to achieve precise identification through complementarity. In addition, improvements in analytical instrumentation techniques have advanced the research process for MAAs.

## 5. Functions of MAAS

### 5.1. Health-Related Functions

#### 5.1.1. Anti-UV

There is a clear positive correlation between the concentration of MAAs in both photosynthetic organisms and their symbionts, and the total radiation intensity they receive in the wild, which further highlights the photoprotective function of MAAs. MAAs have been shown to effectively absorb UVR and dissipate the absorbed energy, thus preventing the formation of reactive oxygen molecules that can result in oxygen stress. As a result, MAAs can act as a natural and safe UV-absorbing filter with a high sun protection factor (SPF) and biological effectiveness protection factor (BEPF), which maintains its stability between pH 2.4 and 5.8 for up to 5 h [49]. In addition, studies have shown that MAAs such as asterina-330, shinorine, mycosporine-glycine, and porphyra-334 can play a significant photoprotective role in cyanobacteria in alpine lakes [120] and *Microcystis aeruginosa* [121]. Exactly these compounds play an important biological role in the ecosystem. For instance, when the dorsal skin of hairless mice was treated with porphyra-334 followed by SPF 3.71, critical wavelength 357–358 nm, no clinical or histopathological skin changes were observed [122]. This evidence suggests that MAAs are an excellent weapon against UVR, as they are natural and effective UV-absorbing compounds that can effectively prevent damage from high UVR [123,124,125].

##### Promoting Cell Proliferation

In a study of human fibroblasts irradiated with UVR, it was found that MAAs have a proliferative effect on human skin fibroblasts. Mycosporine-glycine was the most active and porphyra-334 the least active in inducing cell proliferation [126]. Fernandes further found that shinorine, porphyra-334, and mycosporine-glycine did not cause any toxicity in mouse fibroblasts and could be used to incubate cells [127]. Similarly, when MAAs were added to human TIG-114 fibroblasts for 48 h at concentrations between 0–100 μM, no toxicity was observed and the MAAs acted as a cell proliferator [126].

##### Activating NRF-2

MAAs have been shown to have a range of biological activities in the human system. The Keap1-Nrf2 pathway is an important regulator of cellular oxidative stress, as recognition of ROS by Keap1 leads to the release of Nrf2 and the subsequent activation of cytoprotective genes [128]. When exposed to UVR, Keap2 proteins at the Nrf1 binding site can have competitive interactions, and porphyra-334 and shinorine can then act as antagonists, inhibiting Keap1-Nrf2 binding and increasing the release of Nrf2. This results in the upregulation of the activation of cytoprotective genes under oxidative stress conditions [123,129]. Therefore, shinorine and porphyra-334 are effective activators of the cytoprotective Keap1-Nrf2 pathway under UV-induced stress conditions.

##### Protecting against DNA Damage

Direct and indirect toxic effects of UVR on DNA molecules can mediate photoaging. Absorption of UVB photons by DNA can lead to the production of pyrimidine dimers, resulting in DNA strand defects [130]. MAAs, which absorb UV wavelengths, protect cells from DNA damage [95,131]. Porphyra-334 has been found to be viable for human keratin-forming cell protection and can reduce DNA damage and dimer formation in fibroblasts (IMR-90) [95,132]. With respect to UVA-mediated damage, porphyra-334 has been observed to inhibit the production of ROS and the expression of MMPs following UVA irradiation and protect cells [114,133]. When MAA-containing Helioguard 365 was used to treat HaCaT cells, an increase in cell survival was observed. Moreover, when human fibroblasts were treated with Helioguard 365, a reduction in the number of cells exposed to UVR-induced DNA damage was noted when MAA-containing sunscreen was used under UVR conditions [134]. Additionally, the addition of Helioguard 365 at a 2% level to a sunscreen with an SPF of 7.2 increased the SPF from 7.2 to 8.2. A 5% concentration of Helioguard 365 was found to reduce UVA-induced DNA damage by 73%.

#### 5.1.2. Anti-Oxidation

Oxidative stress (OS) is a state in which there is an imbalance between the oxidative and anti-oxidation effects in the body, resulting in a negative effect of free radicals. MAAs have been reported to possess powerful anti-oxidation properties, including scavenging reactive oxygen species (ROS), water-soluble free radical assays, and the inhibition of β-carotene oxidation, among others [13,29,71,125,135,136,137]. In one study, the anti-oxidation activity of MAAs in three red algae and marine lichen was measured. It was found that the anti-oxidation activity of the studied MAAs was dose-dependent and increased with the alkalinity of the medium, with the highest anti-oxidation activity at pH 8.5. Porphyra-334 and shinorine were found to exhibit lower water-soluble radical scavenging, whereas asterina-330 demonstrated higher inhibition of β-carotene oxidation compared to vitamin E [29]. These results indicate the superiority of MAAs compared to strong antioxidants.

Mycosporine-glycine has been demonstrated to elicit anti-oxidation effects on LPS-stimulated RAW2.120 macrophages, as evidenced by its ability to downregulate the expression of Sod7, Cat, and Nrf1 [138]. Furthermore, RNA sequencing analysis following porphyra-334 treatment of human follicular dermal papilla (HFDP) cells verified that it could positively regulate MT-related gene expression through its anti-oxidation effect [130]. Similarly, other types of MAAs have been observed to reduce UVA-induced intracellular ROS production in human skin fibroblasts [125]. Various studies have indicated that MAAs are capable of scavenging ROS, inhibiting damage caused by singlet oxygen, and exerting anti-oxidation effects through filtering, and direct and indirect burst mechanisms [139,140,141]. This function has been demonstrated in a variety of MAAs [129,130,136,138,142,143].

#### 5.1.3. Anti-Inflammation

UVR damage can generate ROS, leading to inflammation and immune stress [144]. ROS can act as signaling molecules, inducing oxidative stress and the oxidation of proteins, which in turn activates inflammatory pathways and processes [13,145]. Research indicated that mycosporine-glycine could suppress the expression of the COX-2 gene, thereby reducing inflammation in the human fibroblast HaCaT line [114]. In addition, mycosporine-2-glycine lessened the lipopolysaccharide (LPS)-induced production of the inflammatory signaling factor nitric oxide (NO), and was 2–3 times more powerful than shinorine, porphyra-334, and palythine. Transcriptional analysis revealed that mycosporine-2-glycine significantly inhibited the expression of inducible nitric oxide synthase (iNOS) and COX-2, and hindered the production of inflammatory mediators by inhibiting the nuclear factor-κB (NF-κB) pathway [138]. Similarly, Becker et al. [20] observed that shinorine and porphyra-334 affected the activity of the receptor activator of NF-κB in human cells. NF-κB activity was found to increase with increasing shinorine concentration, demonstrating dose-dependent effects. Consequently, marine algal metabolites may be an effective strategy for the management or prevention of inflammation.

#### 5.1.4. Anti-Aging

Prolonged exposure to UVR and the degradation of collagen fibers and elastin can contribute to the aging of skin. It has been observed that UVR potently inhibits the expression of genes encoding elastin and procollagen C-endopeptidase (PCOLCE). To explore the anti-aging properties of porphyra-334 and shinorine, Kim et al. [146] used human fibroblasts cells, HaCaT cells, and HFDP cells from normal human scalp hair follicles. Their findings demonstrated an improvement of periorbital wrinkles, promotion of collagen formation, and stimulation of cell reproduction. Rui et al. [123] further investigated these effects in ICR mice and observed that MAAs can inhibit both collagen and elastin degradation, suggesting its potential as an effective treatment for aging skin. Porphyra-334 also inhibited the production of ROS, as well as the expression of matrix metalloproteinases (MMPs), while increasing the levels of procollagen, type I collagen, and elastin [133]. Moreover, the UV-absorbing compounds mycosporine-glycine, shinorine, and porphyra-334 have been found to regulate the expression of genes associated with UV-induced oxidative stress, inflammation, and skin aging [114]. Tarasuntisuk [147] isolated endotoxin-free mycosporine-2-glycine, which has greater protein cross-linking inhibitory activity than the inhibitor aminoguanidine, and showed that this compound can inhibit the formation of advanced glycation end products (AGEs) and excess collagenase activity. Orfanoudaki [148] assessed the collagenase inhibitory activity of multiple MAAs in vitro using spectrofluorimetry and found that most MAAs had significant inhibition of collagenase and could slow the rate of aging. Therefore, in this era of maintenance-oriented anti-aging, MAAs may offer great promise in combatting the progression of irreversible aging.

#### 5.1.5. Anti-Cancer

Recently, the anti-proliferation activity of MAAs has been demonstrated in tumor cells. Shinorine and porphyra-334 were used to treat mouse cutaneous melanoma cells, and it was found that both MAAs exerted a significant inhibition of the cells’ proliferation in a dose-dependent manner [149]. These findings support the role of MAAs in anti-tumor activities. Extracts of usujirene, palythine, and asterina-330 have been shown to induce apoptosis in HeLa cells, and display both anti-proliferation and anti-cancer effects [150]. Exposure to UVR leads to the formation of covalent bonds between adjacent pyrimidine bases in DNA, resulting in the formation of pyrimidine dimers, and causing faulty DNA replication and abnormal gene expression or mutations. MAAs have been found to block the production of UV-induced DNA damage, leading to reductions of 18.9% and 41.1% in T<>T pyrimidine-pyrimidone 6-4 dimers and cyclobutane cis-syn T<>T 5-6 dimers, respectively, in *Porphyra yezoensis* cells, thus providing protection for the DNA molecules and inhibiting cancer development [95]. This natural, green active substance has the potential to be an important component in the future development of anti-cancer therapeutic agents and applications.

#### 5.1.6. Promoting Wound Healing

Shinorine, mycosporine-glycine, and porphyra-334 had a significant impact on the wound healing process in human keratinocytes by stimulating focal adhesion kinases (FAKs), extracellular signaling regulatory kinases (ERKs), and c-Jun N-terminal kinases (JNKs) [151]. This demonstrates that MAAs can be effectively employed as a novel wound-healing agent and are likely to be an innovative biomaterial for wound-healing therapy.

#### 5.1.7. Preventing Abiotic Stress

Records have indicated that MAAs can improve cellular resistance to abiotic stressors such as salinity, humidity, and temperature [21,152]. A high-salt environment can trigger cellular dehydration and an increase in ROS, resulting in the generation of oxidative stress. MAAs in this environment are known as ‘osmotic solutes’ or ‘compatible solutes’, and are abundant in the organism, providing osmotic balance to the cells [21], thus reducing the impact of salt stress in hypertonic environments. The high concentration of MAAs in algae, combined with its amphiphilic or acidic nature, suggests that MAAs may also regulate infiltration in cyanobacterial cells [153]. Freshwater organisms also tend to accumulate these substances, albeit in much smaller concentrations. It has been found that MAAs in organisms living in cold zones can also act as osmoprotectants in cold environments [152,154]. Furthermore, MAAs play a significant role in mitigating the effects caused by elevated temperatures.

#### 5.1.8. Regulating Embryonic Development and Biological Growth

MAAs have been found to have protective effects on embryonic and larval development [154]. For example, they have been observed to have protective effects on lumbar flagellate and sea urchin embryos, although the exact target of such protection is not yet fully understood. Additionally, MAAs have been reported to be associated with the growth of some organisms [155,156], yet the underlying regulatory mechanisms remain to be elucidated.

### 5.2. Other Functions

#### 5.2.1. Protecting against Coral Bleaching

MAAs are thermally unstable and their UV protection is relatively weak at high latitudes, elucidating the synergistic effect of high-intensity UVR and temperature in coral bleaching [157,158]. Generally, the biosynthesis of MAAs under multiple abiotic stresses necessitates materials, energy, and a balance between other metabolic requirements, and their role in protecting against coral bleaching warrants further validation [159]. However, there is a dearth of in-depth investigations into the protective effects of MAAs against coral bleaching, making it a potential avenue for ecological conservation.

#### 5.2.2. Storing Nitrogen

**Figure 4:**
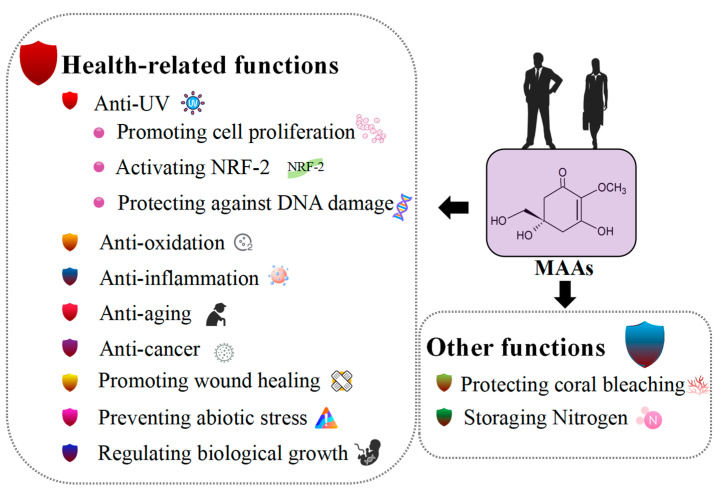
Functional roles of MAAs.

MAAs have been identified as intracellular reservoirs of nitrogen [21,42]. These molecules can contain single or multiple nitrogen atoms, which can be released through appropriate mechanisms when necessary [21,160]. The functional roles of MAAs are shown in Figure 4.

## 6. Future Outlook

Increased human activities have caused a rise in damage to the atmospheric ozone layer, leading to more intense UVR reaching the Earth’s surface. MAAs have been widely studied and discovered as natural UV-absorbing compounds in a variety of algae, bacteria, fungi, zooplankton, and plants. Due to their unique molecular structure and properties, MAAs are very soluble in both water and various organic solvents, yet their content in living organisms is comparatively small, making extraction and purification of high-purity MAAs quite challenging. On the one hand, with a comprehensive understanding of the MAAs’ biosynthesis pathway, large-scale production by heterologous expression is becoming a reality; on the other hand, the development of efficient and convenient methods for the extraction and purification of high-purity MAAs from algal resources is an urgent technical bottleneck that needs to be addressed.

The study of the occurrence and physiological functions of MAAs can help us to better understand the UV protection mechanisms in organisms. Several questions still remain unanswered, such as the localization of MAAs in intracellular chloroplasts or other UV-sensitive sites, the potential of MAAs as chemical signaling molecules or other physiological regulators, and the molecular mechanisms of UV signal transduction. It would also be beneficial to introduce the biosynthetic pathways of MAAs into higher plants (e.g., the flavonoid-deficient mutant of Arabidopsis) in order to gain insight into the biomolecular evolution of UV protection. The major success of Helioguard 365, a commercially available naturally active sunscreen made from MAAs, is an encouraging development in the field of MAA research. However, the absence of a commercially available standard product has made it difficult for teams actively exploring MAAs. Therefore, future research should focus on better regulating the biosynthesis and production of MAAs to lay the foundation for the production of personalized natural sunscreens. Although the road ahead is long, with sufficient effort and perseverance we will eventually overcome the obstacles and apply MAAs to a variety of applications in the sunscreen industry and other biotechnologies.

## Figures and Tables

**Table 1:** Types of MAAs.

Type	Name	Maximum Absorbance	*m/z*	Source	Reference
1	Bostrychines	/	/	Rhodophyta	[59]
2	Mycosporine-glutomicol	/	/	Fungi	[60]
3	Mycosporine-glutominol	/	/	Fungi	[61]
4	Mycosporine-glutamicol ethyl ester	/	/	Fungi, Lichen	[53]
5	Mycosporine-glutaminol	/	/	Cyanobacteria, Fungi, Lichen	[61]
6	Mycosporine-serinol	/	/	Fungi, Cyanobacteria	[62]
7	Catenelline	/	/	Rhodophyta	[28]
8	Normycosporine-glutamic acid	/	/	Fungi	[63]
9	Mycosporine-glutamicol	/	/	Fungi	[63]
10	Mycosporine-glutamine	/	/	Fungi	[63]
11	Mycosporine-hydroxyglutamicol	/	/	Lichen	[64]
12	Deoxygadusol	268	189.0757	Cnidaria, Echinodermata, Lichen, Chordata	[6]
13	Gadusol	294	205.0707	Arthropoda, Chordata, Lichen	[6]
14	Mycosporine-taurine	309	296.0798	Cnidaria, Cyanobacteria, Lichen	[65]
15	Mycosporine-tau	309	318	Cyanobacteria	[66]
16	Mycosporine-glycine	310	246.0972	Arthropoda, Chlorophyta, Chordata, Cnidaria, Cyanobacteria, Dinoflagellata, Echinodermata, Lichen, Miozoa, Mollusca, Ochrophyta, Phaeophyta, Porifera, Rhodophyta	[60,67,68]
17	Mycosporine-serine sulfateinol	310	/	Aquatic Organisms	[51]
18	Mycosporine-glutamine	310	/	Fungi	[51]
19	Mycosporine-ornithine	310	303.1551	Cyanobacteria	[69]
20	Mycosporine-alanine	310	/	Cyanobacteria, Fungi	[70]
21	Mycosporine-β-alanine	310	/	Cnidaria	[71]
22	Mycosporine-GABA	310	/	Cyanobacteria, Dinoflagellata	[71,72]
23	Mycosporine-ornithine	310	303.2	Cyanobacteria	[73]
24	Mycosporine-serine	310	/	Fungi	[71]
25	Mycosporine hydroxyglutamicol	310	320.1345	Lichen	[64]
26	Mycosporine-lysine	310	317.2	Cyanobacteria	[73]
27	273-DA MAA	310	/	Cyanobacteria	[72]
28	Collemin A	311	/	Lichen	[74]
29	1050-DA MAA	312	/	Cyanobacteria	[75]
30	Palythine-Thr-sulfate	320	/	Cnidaria	[76]
31	Palythine-Ser-sulfate	320	/	Cnidaria	[76]
32	Palythine	320	245.1132	Rhodophyta	[76]
33	Palythine-serine-sulfate	320	/	Cnidaria	[76]
34	Palythine-serine	320	275.1238	Cnidaria, Cyanobacteria	[68,68]
35	Hexose-bound palythine-serine	320	437.1766	Cyanobacteria	[69]
36	Palythine-serine sulfate	321	355.0806	Lichen	[6]
37	Palythine-threonine	321	289.1394	Cnidaria, Cyanobacteria	[69]
38	Palythine-threonine sulfate	321	369.0962	Cnidaria	[6]
39	Hexose-bound palythine-threonine	322	451.1896	Cyanobacteria	[69]
40	Palythine-glutamine	322	316.1472	Rhodophyta	[77]
41	Palythine-glutamic acid	322	317.131	Rhodophyta	[77]
42	612-DA MAA	322	/	Cyanobacteria	[78]
43	Prasiolin	324	/	Chlorophyta	[79]
44	Klebsormidin A	324	468.1699	Charophyta	[80]
45	Klebsormidin B	324	306.1160	Charophyta	[80]
46	Mycosporine-methylamine-serine	327	289.1394	Cnidaria	[65,67]
47	Mycosporine-methylamine-threonine	327	303.1551	Cnidaria, Rhodophyta	[65,67]
48	Mycosporine-glycine-glutamic acid	330	375.1398	Cnidaria, Lichen, Rhodophyta	[65]
49	Asterina-330	330	289.1394	Arthropoda, Chlorophyta, Chordata, Echinodermata, Lichen, Mollusca, Ochrophyta, Phaeophyta, Rhodophyta	[65]
50	*N*-Methylpalythine	330	259.1288	Cyanobacteria	[69]
51	Asterina-methyl ester	330	303	Dinoflagellata	[81]
52	Aplysiapalythine C	330	/	Cyanobacteria, Mollusca	[71]
53	Mycosporine-serine-glycine methyl ester	331	347	Dinoflagellata	[82]
54	880-DA MAA	331	/	Cyanobacteria	[72]
55	Mycosporine-threonine-β-alanine	332	361.1586	Rhodophyta	[77]
56	Palythinol	332	303.1551	Chlorophyta, Lichen, Phaeophyta, Porifera, Rhodophyta	[65,60]
57	Mycosporine-glycine-aspartic acid	332	/	Arthropoda	[71]
58	Aplysiapalythine A	332	/	Mollusca, Rhodophyta	[71]
59	Aplysiapalythine B	332	/	Mollusca, Rhodophyta	[71]
60	4-Deoxygadusol	333	/	Cnidaria, Echinodermata, Lichen, Chordata	[83]
61	Mycosporine-threamine-glutamic acid	333	375.1737	Rhodophyta	[77]
62	Mycosporine-2-glycine	334	303.1187	Bacillariophyta, Cnidaria, Cyanobacteria, Echinodermata, Mollusca, Rhodophyta	[65]
63	Shinorine	334	333.1292	Arthropoda, Bacillariophyta, Chlorophyta, Chordata, Cyanobacteria, Dinoflagellata, Lichen, Miozoa, Mollusca, Ochrophyta, Phaeophyta, Porifera, Rhodophyta	[67,68]
64	Porphyra-334	334	347.1449	Arthropoda, Bacillariophyta, Chlorophyta, Chordata, Cnidaria, Cyanobacteria, Dinoflagellata, Miozoa, Mollusca, Ochrophyta, Phaeophyta, Porifera, Rhodophyta	[65,67]
65	Aplysiapalythine D	334	259.1244	Cyanobacteria	[84]
66	508-DA MAA	334	/	Cyanobacteria	[78]
67	13-O-(β-galactosyl)-porphyra-334	334	/	Cyanobacteria	[55]
68	Mycosporine-glycine-valine	335	345.1556	Arthropoda, Cnidaria, Chordata, Echinodermata, Lichen, Mollusca, Porifera	[65]
69	Mycosporine-threonine-glutamine	335	418.1739	Rhodophyta	[77]
70	478-DA MAA	335	/	Fungi	[75]
71	*E*-palythenic acid	337	329.1343	Aquatic Organisms	[4]
72	*Z*-palythenic acid	337	/	Aquatic Organisms	[4]
73	Mycosporine-threonine-glutamic acid	337	419.1172	Rhodophyta	[77]
74	M-343	343	387	Cyanobacteria	[66]
75	Dehydroxyl-usujirene	356	268	Cyanobacteria	[66]
76	Usujirene	357	285.1445	Cyanobacteria	[65]
77	Palythene	360	/	Arthropoda, Chlorophyta, Cnidaria, Lichen, Mollusca, Phaeophyta, Porifera, Rhodophyta	[68]
78	Euhalothece-362	362	331.1500	Cyanobacteria	[68]

*/* No relevant information.

**Table 3:** The detection methods for MAAs.

Reagent	Detection Method	Organism Analyzed	Substance Detected	Reference
Ammonium hydroxide; Methanol; Acetonitrile	HPLC	Coral, Sea anemone, Microalgae, Phytoplankton	Palythine, Mycosporine-2-glycine, Shinorine, Usujirene, Palythene	[119]
Aqueous acetic acid; MeOH	LC-MS	Cyanobacterial, Actinomycete, Fungi	Mycosporine glutaminol, Mycosporine-serinol, Mycosporine-alanine, Mycosporine-serine, Palythine, Asterina-330, Palythinol, Porphyra-334	[22]
/	LC-MS-MS	Phytoplankton, Pteropod	Mycosporine-glycine, Palythine, Shinorine, Porphyra-334	[115]
Methanol; Ethanol	HPLC-DAD, HPLC-ESI-MS	Red macroalgae	Palythine, Shinorine, Porphyra-334	[23]
Formic acid; Methanol	NMR, HRMS	Bostrychia, Scorpioides	Mycosporine-glutamicol, Palythine-glutamine, Palythine-glutamic acid, Mycosporine-threonine-β-alanine, Mycosporine-threamine-glutamic acid, Mycosporine-threonine-glutamine, Mycosporine-threonine-glutamic acid	[77]
Methanol; Formic acid; Acetic acid	HPLC-DAD	Red macroalgae	Bostrychines	[59]
Ammonium acetate buffer; Acetonitrile; Ethyl acetate	LC-MS	Red macroalgae	Palythine, Asterina-330, Shinorine, Porphyra-334, Usujirene, Palythene	[40]
Formic acid; Methanol; Formic acid	LC-MS, NMR	Red macroalgae	Palythine, Asterina-330, Shinorine, Porphyra-334	[37]
Ammonium formate; Acetonitrile	QTOFMS	Cyanobacteria	Mycosporine-glycine, Mycosporine-ornithine, Palythine-serine, Hexose-bound palythine-serine, Palythine-threonine, Hexose-bound palythine-threonine, Palythinol, *N*-Methylpalythine	[69]

*/* No relevant information.

## Data Availability

Data will be made available on request.
